# Gene model for the ortholog of *eIF4E1* in *Drosophila yakuba*

**DOI:** 10.17912/micropub.biology.001020

**Published:** 2025-01-29

**Authors:** Bailey Lose, Jeremy Girard, Josephine Hayes, Lane Weast, Natalie Minkovsky, Sarah Justice, Jack A. Vincent, James J. Youngblom, Lindsey J. Long, Chinmay P. Rele, Laura K Reed

**Affiliations:** 1 University of Alabama, Tuscaloosa, AL USA; 2 California State University Stanislaus, Turlock, CA USA; 3 Oklahoma Christian University, Edmond, OK USA; 4 Community College of Baltimore County, Baltimore, MD USA; 5 Taylor University, Upland, IN USA; 6 University of Washington - Tacoma, Tacoma, WA USA

## Abstract

Gene model for the ortholog of *eukaryotic translation initiation factor 4E1* ( *eIF4E1* ) in the Dyak_CAF1 Genome Assembly (GenBank Accession: GCA_000005975.1 ) of *Drosophila yakuba* . This ortholog was characterized as part of a developing dataset to study the evolution of the Insulin/insulin-like growth factor signaling pathway (IIS) across the genus *Drosophila* using the Genomics Education Partnership gene annotation protocol for Course-based Undergraduate Research Experiences.

**Figure 1. Genomic neighborhood and gene model for eIF4E1 in D. yakuba:**
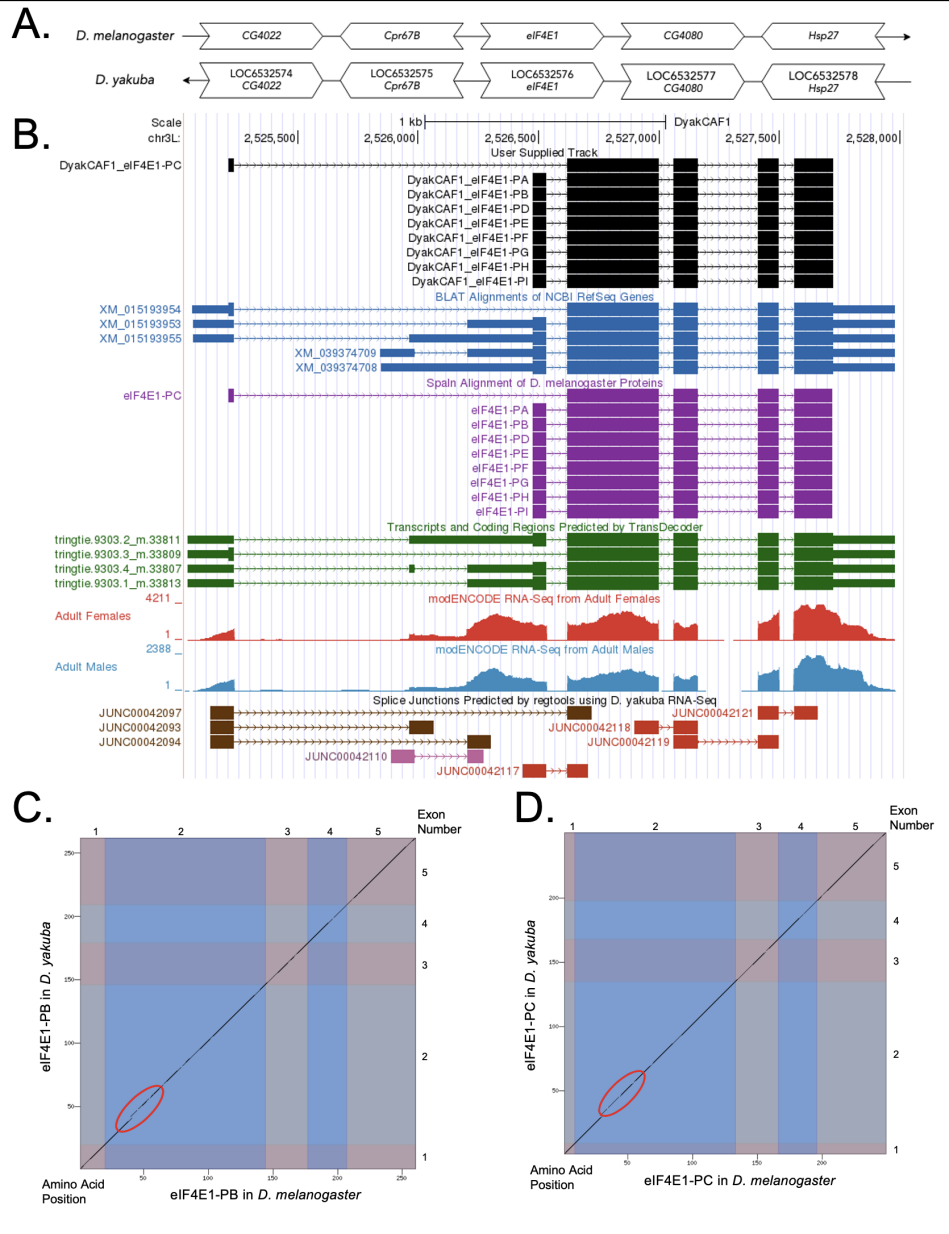
**
(A) Synteny comparison of the genomic neighborhoods for *eIF4E1* in *Drosophila melanogaster* and *D. yakuba* .** Thin underlying arrows indicate the DNA strand within which the reference gene– *eIF4E1* –is located in *D. melanogaster* (top) and *D. yakuba* (bottom) genomes. The thin arrow pointing to the right indicates that *eIF4E1* is on the positive (+) strand in *D. melanogaster* , and the thin arrow pointing to the left indicates that *eIF4E1* is on the negative (-) strand in *D. yakuba* The wide gene arrows pointing in the same direction as *eIF4E1* are on the same strand relative to the thin underlying arrows, while wide gene arrows pointing in the opposite direction of *eIF4E1* are on the opposite strand relative to the thin underlying arrows. White gene arrows in *D. yakuba* indicate orthology to the corresponding gene in *D. melanogaster* . Gene symbols given in the *D. yakuba* gene arrows indicate the orthologous gene in *D. melanogaster* , while the locus identifiers are specific to *D. yakuba* . **(B) Gene Model in GEP UCSC Track Data Hub (Raney et al., 2014).** The coding-regions of *eIF4E1* in *D. yakuba* are displayed in the User Supplied Track (black); CDS are depicted by thick rectangles and introns by thin lines with arrows indicating the direction of transcription. Subsequent evidence tracks include BLAT Alignments of NCBI RefSeq Genes (dark blue, alignment of Ref-Seq genes for *D. yakuba* ), Spaln of *D. melanogaster* Proteins (purple, alignment of Ref-Seq proteins from *D. melanogaster* ), Transcripts and Coding Regions Predicted by TransDecoder (dark green), RNA-Seq from Adult Females and Adult Males (red and light blue, respectively; alignment of Illumina RNA-Seq reads from *D. yakuba* ), and Splice Junctions Predicted by regtools using *D. yakuba* RNA-Seq (SRP006203 - Graveley et al, 2010). Splice junctions shown have a read-depth of 232, 500-999 and >1000 supporting reads in pink, brown and red, respectively. **(C) Dot Plot of eIF4E1-PB in *D. melanogaster* ( *x* -axis) vs. the orthologous peptide in *D. yakuba* ( *y* -axis).** Amino acid number is indicated along the left and bottom; CDS number is indicated along the top and right, and CDS are also highlighted with alternating colors. A region with a reduced sequence similarity is circled in red. **(D) Dot Plot of eIF4E1-PC in *D. melanogaster* (x-axis) vs. the orthologous peptide in *D. yakuba* (y-axis).** A region with reduced sequence similarity is circled in red.

## Description

**Table:** 

*This article reports a predicted gene model generated by undergraduate work using a structured gene model annotation protocol defined by the Genomics Education Partnership (GEP; thegep.org) for Course-based Undergraduate Research Experience (CURE). The following information in this box may be repeated in other articles submitted by participants using the same GEP CURE protocol for annotating Drosophila species orthologs of Drosophila melanogaster genes in the insulin signaling pathway.* "In this GEP CURE protocol students use web-based tools to manually annotate genes in non-model *Drosophila* species based on orthology to genes in the well-annotated model organism fruitfly *Drosophila melanogaster* . The GEP uses web-based tools to allow undergraduates to participate in course-based research by generating manual annotations of genes in non-model species (Rele et al., 2023) . Computational-based gene predictions in any organism are often improved by careful manual annotation and curation, allowing for more accurate analyses of gene and genome evolution (Mudge and Harrow 2016; Tello-Ruiz et al., 2019) . These models of orthologous genes across species, such as the one presented here, then provide a reliable basis for further evolutionary genomic analyses when made available to the scientific community.” (Myers et al., 2024) . “The particular gene ortholog described here was characterized as part of a developing dataset to study the evolution of the Insulin/insulin-like growth factor signaling pathway (IIS) across the genus *Drosophila* . The Insulin/insulin-like growth factor signaling pathway (IIS) is a highly conserved signaling pathway in animals and is central to mediating organismal responses to nutrients (Hietakangas and Cohen 2009; Grewal 2009) .” (Myers et al., 2024) . “ *D. yakuba (* NCBI:txid7245) is part of the *melanogaster* species group within the subgenus *Sophophora* of the genus *Drosophila* ( Sturtevant 1939 ; Bock and Wheeler 1972 ). It was first described by Burla (1954). *D. yakuba* is wide-spread in sub-Saharan Africa and Madagascar (Lemeunier et al., 1986; https://www.taxodros.uzh.ch , accessed 1 Feb 2023; Markow and O'Grady 2005) where figs served as a primary host along with other rotting fruits ( Lachaise and Tsacas 1983 ) *.* ” (Koehler et al., 2024) .

We propose a gene model for the *D. yakuba* ortholog of the *D. melanogaster* eukaryotic translation initiation factor 4E1 ( *eIF4E1* ) gene. The genomic region of the ortholog corresponds to the uncharacterized protein LOC6532576 (RefSeq accession XP_015049441.1 ) in the Dyak_CAF1 Genome Assembly of *D. yakuba* (GenBank Accession: GCA_000005975.1 ; *Drosophila* 12 Genomes Consortium et al., 2007). This model is based on RNA-Seq data from *D. yakuba* ( SRP006203 ; Graveley et al., 2011) and *eIF4E1* in *D. melanogaster* using FlyBase release FB2022_04 ( GCA_000001215.4 ; Larkin et al., 2021; Gramates et al., 2022; Jenkins et al., 2022).

*eukaryotic translation initiation factor 4E1* ( *eIF4E1* ) encodes eIF4F cap-binding complex essential cap-dependent translation of mRNA, and binds the 7-methyl-guanosine cap structure of mRNA in *Drosophila* (Lachance et al., 2002; Lavoie et al., 1996) . The protein product of *eIF4E-3* , a paralog of *eIF4E1* , is specifically required during spermatogenesis in *Drosophila* (Hernendez et al., 2012).

***Synteny***

The reference gene, *eIF4E1* , occurs on chromosome 3L in *D. melanogaster* and is flanked upstream by *CG4022* and *Cuticular protein 67B* ( *Cpr67b* ) and downstream by *CG4080* and *Heat shock protein 27* ( *Hsp27* ). The *tblastn* search of *D. melanogaster* eIF4E1-PB (query) against the *D. yakuba* (GenBank Accession: GCA_000005975.1 ) Genome Assembly (database) placed the putative ortholog of *eIF4E1* within scaffold chromosome 3L (CM000159.2) at locus LOC6532576 ( XP_015049441.1 ) with an E-value of 1e-77 and a percent identity of 65.56%. Furthermore, the putative ortholog is flanked upstream by LOC6532574 ( XP_015049438.1 ) and LOC6532575 ( XP_002093319.1 ) which correspond to *CG4022* and *Cpr67b* in *D. melanogaster* (E-value: 0.0 and 7e-170; identity: 90.08% and 98.46%, respectively, as determined by *blastp* ; Figure 1A, A ltschul et al., 1990). The putative ortholog *eIF4E1* is flanked downstream by LOC6532577 ( XP_015049442.1 ) and LOC6532578 ( XP_002093322.1 ) which correspond to *CG4080* and *Hsp27* in *D. melanogaster* (E-value: 0.0 and 2e-132; identity: 96.63% and 89.72%, respectively, as determined by *blastp* ). The putative ortholog assignment for *eIF4E1* in *D. yakuba* is supported by the following evidence: The genes surrounding the *eIF4E1* ortholog are orthologous to the genes at the same locus in *D. melanogaster* and local synteny is completely conserved, supported by e-values and percent identities, so we conclude that LOC6532576 is the correct ortholog of *eIF4E1* in *D. yakuba* ( Figure 1A ).

***Protein Model***

*eIF4E1* in *D. yakuba* has two unique protein-coding isoforms eIF4E1-PB (identical to eIF4E1-PA, eIF4E1-PD, eIF4E1-PE, eIF4E1-PF, eIF4E1-PG, eIF4E1-PH, eIF4E1-PI) and eIF4E1-PC ( Figure 1B ). mRNA isoforms *eIF4E1-RB* ( *eIF4E1-RA* , *eIF4E1-RD* , *eIF4E1-RE* , *eIF4E1-RF* , *eIF4E1-RG* , *eIF4E1-RH* , *eIF4E1-RI* ) and *eIF4E1-RC* contain five CDSs. Relative to the ortholog in *D. melanogaster* , the RNA CDS number and protein isoform count is conserved. The sequence of eIF4E1-PB in *D. yakuba* has 93.49% identity (E-value: 1e-142) with the protein-coding isoform eIF4E1-PB in *D. melanogaster* , as determined by *blastp* ( Figure 1C ). Minor gaps in the dot plots of eIF4E1-PB ( Figure 1C ) and eIF4E1-PC ( Figure 1D ) represent a region of lower sequence similarity, highlighted by red circles, including a short indel of two amino acids in the second exon of both isoforms. Coordinates of this curated gene model are stored by NCBI at GenBank/BankIt (accession **BK059542 , BK059543 , BK059544 , BK059545 , BK059546 , BK059547 , BK059548 , BK059549** and **BK059550 )** . These data are also archived in the CaltechDATA repository (see “Extended Data” section below).

## Methods

Detailed methods including algorithms, database versions, and citations for the complete annotation process can be found in Rele et al. (2023). Briefly, students use the GEP instance of the UCSC Genome Browser v.435 ( https://gander.wustl.edu ; Kent WJ et al., 2002; Navarro Gonzalez et al., 2021) to examine the genomic neighborhood of their reference IIS gene in the *D. melanogaster* genome assembly (Aug. 2014; BDGP Release 6 + ISO1 MT/dm6). Students then retrieve the protein sequence for the *D. melanogaster* reference gene for a given isoform and run it using *tblastn* against their target *Drosophila* species genome assembly on the NCBI BLAST server ( https://blast.ncbi.nlm.nih.gov/Blast.cgi ; Altschul et al., 1990) to identify potential orthologs. To validate the potential ortholog, students compare the local genomic neighborhood of their potential ortholog with the genomic neighborhood of their reference gene in *D. melanogaster* . This local synteny analysis includes at minimum the two upstream and downstream genes relative to their putative ortholog. They also explore other sets of genomic evidence using multiple alignment tracks in the Genome Browser, including *BLAT* alignments of *RefSeq* Genes, *Spaln* alignment of *D. melanogaster* proteins, multiple gene prediction tracks (e.g., *GeMoMa, Geneid, Augustus* ), and *modENCODE* RNA-Seq from the target species. Detailed explanation of how these lines of genomic evidenced are leveraged by students in gene model development are described in Rele et al. (2023). Genomic structure information (e.g., CDSs, intron-exon number and boundaries, number of isoforms) for the *D. melanogaster* reference gene is retrieved through the Gene Record Finder ( https://gander.wustl.edu/~wilson/dmelgenerecord/index.html ; Rele et al *.,* 2023). Approximate splice sites within the target gene are determined using *tblastn* using the CDSs from the *D. melanogaste* r reference gene. Coordinates of CDSs are then refined by examining aligned modENCODE RNA-Seq data, and by applying paradigms of molecular biology such as identifying canonical splice site sequences and ensuring the maintenance of an open reading frame across hypothesized splice sites. Students then confirm the biological validity of their target gene model using the Gene Model Checker ( https://gander.wustl.edu/~wilson/dmelgenerecord/index.html ; Rele et al., 2023), which compares the structure and translated sequence from their hypothesized target gene model against the *D. melanogaster* reference gene model. At least two independent models for this gene were generated by students under mentorship of their faculty course instructors. These models were then reconciled by a third independent researcher mentored by the project leaders to produce the final model presented here. Note: comparison of 5' and 3' UTR sequence information is not included in this GEP CURE protocol.

## Data Availability

Description: A GFF, FASTA, and PEP of the model. Resource Type: Model. DOI: https://doi.org/10.22002/hx6n6-een12
